# Supplementary material for: Genetic and genomic resources to study natural variation in Brassica rapa
Source: Plant Direct. 2020 Dec 22;4(12):e00285. doi: 10.1002/pld3.285 (PMC7755128; doi:10.1002/pld3.285)
Supplement: Supplementary file 1 — Fig S1 [file PLD3-4-e00285-s001.pdf]

Supplemental Materials for

Genetic and Genomic Resources to Study Natural Variation in *Brassica rapa*

Ping Lou[1], Scott Woody[2], Kathleen Greenham[1,3], Robert VanBuren[4], Marivi Colle[4], Patrick P. Edger[4], Ryan Sartor[5], Yakun Zheng[1,6], Nathan Levendoski[2], Jan Lim[2], Calvin So[2], Brian Stoveken[2], Timothy Woody[2], Jianjun Zhao[6], Shuxing Shen[6], Richard M. Amasino[2], C. Robertson McClung[1]

[1] Department of Biological Sciences, Dartmouth College, Hanover, NH, USA
[2] Department of Biochemistry, University of Wisconsin, Madison, WI, USA
[3] Department of Plant and Microbial Biology, University of Minnesota, St. Paul, MN, USA
[4] Department of Horticulture, Michigan State University, East Lansing, MI, 48824, USA
[5] Crop and Soil Sciences, North Carolina State University, Raleigh, NC, 27695, USA
[6] State Key Laboratory of North China Crop Improvement and Regulation, Collaborative Innovation Center of Vegetable Industry in Hebei, Department of Horticulture, Hebei Agricultural University, Baoding, 071001, China

Ping Lou, Scott Woody, and Kathleen Greenham contributed equally to this work.

Supplemental Materials include:

Supplemental Figure S1. (A) Microsynteny analysis of members of the *PAP* Gene Family in Arabidopsis and *Brassica rapa*. *B. rapa* genes retained after fractionation in each of the genomic regions syntenic to the Arabidopsis PAP genes are indicated. **(B)** Multiple alignment of PAP proteins of *Arabidopsis* and *Brassica rapa.* Amino acid differences between the R500 and L58 PAP(A03) variants are colored, with highly conserved residues in red and dissimilar residues in yellow or cyan. *, :, and . indicates fully, strongly, and weakly conserved, respectively. (**C)** Nucleotide sequence alignment of R500 (upper) and L58 (lower) alleles of the *PAP2(A03)* (BraA03g40160R) gene. Exonic sequences are highlighted in cyan and intronic sequences are not highlighted. Nucleotide polymorphisms are indicated by red, with substitutions indicated by # and indels indicated by filled boxes. Asterisks mark 10 bp intervals.

Supplemental Table S1. (A) Genetic map positions of Single Nucleotide Polymorphism (SNP) Markers and (B) marker inheritance in 121 lines from the R500 x IMB211 Recombinant Inbred Line population (Markelz et al., 2017) used to construct the high-density genetic map onto which the Pilon based contigs were anchored into a chromosome scale assembly of the *B. rapa* R500 genome. (B) Single Nucleotide Polymorphism (SNP) marker inheritance in 121 lines from the R500 x IMB211 Recombinant Inbred Line population (Markelz et al., 2017) used to construct the high-density genetic map onto which the Pilon based contigs were anchored into a chromosome scale assembly of the *B. rapa* R500 genome.

Supplemental Table S2. List of predicted protein-coding genes in *B. rapa* R500 V1.2. Start and end positions for each predicted coding sequence are indicated. Strand indicates the mRNA-like strand of the DNA. Block refers to the syntenic block in the *A. thaliana* genome (Parkin et

al., 2005; Zhang et al., 2018).

Supplemental Table S3. Correspondence of the R500 V1.2 predicted protein-coding gene annotations with the Chiifu V1 gene annotations used in the NCBI and *EnsemblPlants* databases.

Supplemental Table S4. Gene Ontology (GO) terms for genes in the *B. rapa* R500 genome. One or more GO terms were assigned to 38,197 unique gene annotations. The "Gene_ID" field lists *B. rapa* R500 gene accessions. The "GO_ID" field lists the GO term (category) associated with each gene. The "InterPro_ID" field lists accession numbers for functionally characterized InterPro protein domains (see https://www.ebi.ac.uk/interpro). InterPro domains are used to assign GO terms to uncharacterized genes. If no InterPro domain is found, this field has "NA" and the closest Arabidopsis homolog is used to assign GO terms. In this case, the field "At_Homolog" lists the closest Arabidopsis protein match for each gene. The field "Assignment_Type" has an entry of either "InterProScan" or "AtHomology" and specifies which method was used to assign each GO term. The field "GO_Term_Name" lists the short name for each term. The field "GO_Ontology" specifies which of the 3 ontologies (Molecular Function, Biological Process or Cellular Component) each term comes from. The field "GO_Term_Definition" provides a longer description for each term.

Supplemental Table S5. Gene Ontology (GO) Slim terms for genes in the R500 genome. GO Slim is a greatly reduced set of higher level (more broad) Gene Ontology terms. One or more of only 97 GO Slim terms is assigned to 38,199 unique gene annotations. The "Gene_ID" field list R500 gene accessions. The "GO_ID(GOSlim)" field lists the GO Slim term (category) associated with each gene. The field "GO_Term_Name" lists the short name for each term. The field "GO_Ontology" specifies which of the 3 ontologies (Molecular Function, Biological Process or Cellular Component) each term comes from. The field "GO_Term_Definition" provides a longer description for each term.

Supplemental Table S6. Kyoto Encyclopedia of Genes and Genomes (KEGG) orthology-based functional annotations for genes in the *B. rapa* R500 genome. KEGG uses experimentally confirmed protein functions across various organisms to assign function to unknown proteins based on orthology (see https://www.genome.jp/kegg/ for details and descriptions). Each of 12,428 genes are annotated with one of 3,516 KEGG orthology terms. The field "Gene_Name" lists R500 gene accessions. The field "KEGG_ONTOLOGY_NUMBER" lists the KEGG Orthology term associated with each gene. This is an accession for a functional annotation category. The field "KEGG_PATHWAY", when available, lists one or more KEGG Pathway accessions separated by "|". A KEGG pathway is a defined biochemical pathway in which the gene may be involved. The field "KEGG_ENZYME", when available, lists one or more KEGG enzymes. This is a list of potential known enzymes that each gene may encode.

Supplemental Table S7. (A) Genetic map positions of Single Nucleotide Polymorphism (SNP) Markers for 186 lines from the R500 x L58 Advanced Intercross-Recombinant Inbred Line population was used to construct the high-density genetic map shown in **Figure 2**. Seed coat color, measured as average RGB values for an image of 100 seeds, was used to map the QTL as summarized in **Figure 4A** and **Table 2**. Parental values are included last. (B) Genomic positions of Single Nucleotide Polymorphism (SNP) markers selected for construction of the bin-based

genetic map. (C) Original called SNP data from GATK program, without filtering and binning. ** 0: SNP from R500; 2: SNP from L58; 1: Heterozygous SNP; -1:Missing value.

Supplemental Table S8. (A) Amino acid identity among *A. thaliana* and *B. rapa* R500 *PAP* genes. (B) Amino acid polymorphisms in the coding regions of the R500 and L58 alleles of *PAP2(A03)*.

Supplemental File S1. LTR Libraries with chromosomal location noted. File is in Fasta format.

Supplemental File S2. R Code used for seed coat color analysis.

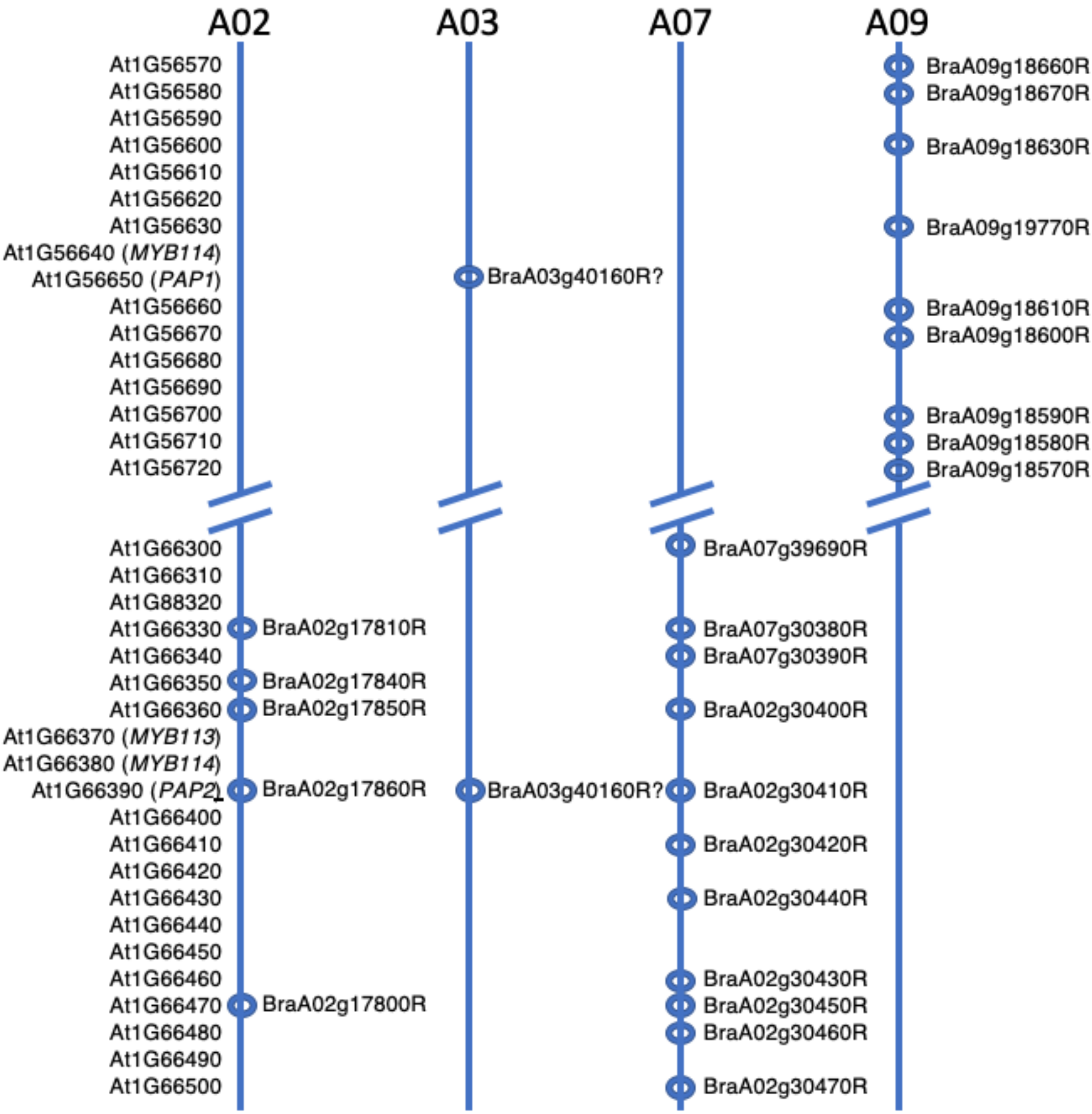

Supplemental Figure S1A. Microsynteny analysis of members of the *PAP* Gene Family in Arabidopsis and *Brassica rapa*.
B. rapa genes retained after fractionation in each of the genomic regions syntenic to the Arabidopsis PAP genes are indicated.

```
Arabidopsis_PAP1            MEGSSKGLRKGAWTTEEDSLLRQCINKYGEGKWHQVPVRAGLNRCRKSCRLRWLNYLKPS  60
Arabidopsis_PAP2            MEGSSKGLRKGAWTAEEDSLLRLCIDKYGEGKWHQVPLRAGLNRCRKSCRLRWLNYLKPS  60
BraA02g17860R_L58_A02       MEGSPKGLRKGAWTAEEDSLLRQCIDKYGEGKWHQVPLRAGLNRCRKSCRLRWLNYLKPS  60
BraA02g17860R_R500_A02      MEGSPKGLRKGAWTAEEDSLLRQCIDKYGEGKWHQVPLRAGLNRCRKSCRLRWLNYLKPS  60
BraA07g30410R_L58_A07       MEGSSQGLKKGAWTAEEDNLLRQCIDKYGEGKWHQVPLRAGLNRCRKSCRLRWLNYLKPS  60
BraA07g30410R_R500_A07      MEGSSQGLKKGAWTAEEDNLLRQCIDKYGEGKWHQVPLRAGLNRCRKSCRLRWLNYLKPS  60
BraA03g40160R_A03_L58       MEDSSKGLTKGAWTAEEDSLLRRCIDKYGEGKWHQVPLRAGLNRCRKSCRLRWLNYLKPN  60
BraA03g40160R_A03_R500      MEDSSKGLTKGAWTAEEDSLLRRCIDKYGEGKWHQIPLRAGLNRCRKSCRLRWLNYLKPT  60
                            **.* :** *****:***.*** **:*********:*:*********************.

Arabidopsis_PAP1            IKRGKLSSDEVDLLLRLHRLLGNRWSLIAGRLPGRTANDVKNYWNTHLSKKHE-PCCKIK  119
Arabidopsis_PAP2            IKRGRLSNDEVDLLLRLHKLLGNRWSLIAGRLPGRTANDVKNYWNTHLSKKHESSCCKSK  120
BraA02g17860R_L58_A02       IKKGKLSSDEVDLLLRLHKLLGNRWSLIAGRLPGRTANDVKNYWNTHLSKKHE-PGCNTK  119
BraA02g17860R_R500_A02      IKKGKLSSDEVDLLLRLHKLLGNRWSLIAGRLPGRTANDVKNYWNTHLSKKHE-PGCNTK  119
BraA07g30410R_L58_A07       IKRGKLNSDEVDLLIRLHKLLGNRWSLIAGRLPGRTANDVKNYWNTHLSKKHE-PGCKTQ  119
BraA07g30410R_R500_A07      IKRGKLNSDEVDLLIRLHKLLGNRWSLIAGRLPGRTANDVKNYWNTHLSKKHE-PGCKTQ  119
BraA03g40160R_A03_L58       IKRGKLSSDEVDLLLRLHKLLGNRWSLIAGRLPGRTANDIKNYWNTHLSKKHE-PCCKTK  119
BraA03g40160R_A03_R500      IKRGKLSSDEVDLLLRLHKLLGNRWSLIAGRLPGRTANDIKNYWNTHLSKKHE-PCCKTK  119
                            **:*:*..******:***:********************:*************   *: :

Arabidopsis_PAP1            MKKRDITPIPTTPALKNNVYKPRPRSFTVNNDCNHLNAPPKVDVNPPCLGLN-INNVCDN  178
Arabidopsis_PAP2            MKKKNIISPPTTPVQKIGVFKPRPRSFSVNNGCSHLNGLPEVDLIPSCLGLK-KNNVCEN  179
BraA02g17860R_L58_A02       MRKRNIPCSSTQPAQKNEVLKPRPRSFTVNNGCSHFNGKPKVDVIPLFLGVNNTNNVCEN  179
BraA02g17860R_R500_A02      MRKRNIPCSSTQPAQKNEVLKPRPRSFTVNNGCSHFNGKPKVDVIPLFLGVNNTNNVCEN  179
BraA07g30410R_L58_A07       MKKRNIPCSYTTPAQKIDVFKPRPRSFTVNSGCSHNNGMPEAGIVPLCLGHNDTNNVSEN  179
BraA07g30410R_R500_A07      MKKRNIPCSYTTPAQKIDVFKPRPRSFTVNSGCSHNNGMPEAGIVPLCLGHNDTNNVSEN  179
BraA03g40160R_A03_L58       MKKRNVTFSSTTPAQKIDVFKPRPRLFTVNNGCSHLHGLPEVDVVPPCLGLNNINNVCEN  179
BraA03g40160R_A03_R500      MKKRNVTFSSTTPAQKIDVFKPRPRLFTVNDGCSHLHGLPEVDVVPPCVGLNNINNVCEN  179
                            *:*:::    * *. *  * ***** *:**..*.* :. *:..: *  :* :  ***.:*

Arabidopsis_PAP1            SIIYNKDKKKDQLVNNLIDGDNMWLEKFLEESQEVDILVPEATTTEKGDTLAFDVDQLWS  238
Arabidopsis_PAP2            SITCNKDDEKDDFVNNLMNGDNMWLENLLGENQEADAIVPEATTAEHGATLAFDVEQLWS  239
BraA02g17860R_L58_A02       SITYKKDAEKYELVNNLMDGENMWWKSLLEESQEPDAIVPESTETEKLATSAFDVEQLWN  239
BraA02g17860R_R500_A02      SITYKKDAEKYELVNNLMDGENMWWKSLLEESQEPDAIVPESTETEKLATSAFDVEQLWN  239
BraA07g30410R_L58_A07       IITCNKDDDKSELVSHLMDGQNRWWESLLDESQDPAALFPETTAIKKGATSAFDVEQLWS  239
BraA07g30410R_R500_A07      IITCNKDDDKSELVSHLMDGQNRWWESLLDESQDPAALFPETTAI---------------  224
BraA03g40160R_A03_L58       SMTCNKAGEKYELYSNLMDGENMWWESLLEESKQPDGLVPKGTATKKGATFAFDVEQLWN  239
BraA03g40160R_A03_R500      SMTCNKAGEKYELFSNLMDGENMWWESLLEQSKQPDGLVPKGTATKKGATFAFDVEQLWN  239
                             :  :*  .* :: .:*::*:* * :.:* :.::   :.*: *

Arabidopsis_PAP1            LFDGETVKFD      248
Arabidopsis_PAP2            LFDGETVELD      249
BraA02g17860R_L58_A02       LLDGETVELD      249
BraA02g17860R_R500_A02      LLDGETVELD      249
BraA07g30410R_L58_A07       LLDGETGT--      247
BraA07g30410R_R500_A07      ----------      224
BraA03g40160R_A03_L58       MLDGETVELD      249
BraA03g40160R_A03_R500      MLDGETVELD      249
```

Supplemental Figure S1B. Multiple alignment of PAP proteins of *Arabidopsis* and *Brassica rapa.* Amino acid differences between the R500 and L58 PAP(A03) variants are colored, with highly conserved residues in red and dissimilar residues in yellow or cyan. *, :, and . indicates fully, strongly, and weakly conserved, respectively.

```
R500 1    ATGGAGGATTCGTCCAAAGGGTTGACCAAAGGTGCATGGACGGCTGAAGAAGACAGTCTCTTGAGGCGATGCATTGATAAGTATGGAGAAGGCAAATGGC 100
L58  1    ATGGAGGATTCGTCCAAAGGGTTGACAAAAGGTGCATGGACCGCTGAAGAAGACAGTCTCTTGAGGCGATGCATTGATAAGTATGGAGAAGGCAAATGGC 100

     101  ATCAAATTCCTTTAAGAGCTGGTATGTTACTTTTTTTTCTTT-TGT-ACACACACAT-------------ATCTGTATACATATAATTAATCACTACGAA 185
     101  ATCAAGTTCCTTTAAGAGCTGGTATGTTACTTTTTTTT-CTT-CTG-CACACACACATATATGTACATATGATCTGTATACATATAATTAATCACTATGAA 197

     186  AAAC-TTCTTTC-TCTTTGTCTTCTACAGTACTACTCGGA-GAATTAATTAACACATGGCTGCACAAAACAAAGTTTTTCTTTTTGTCATAATGAACAAA 282
     198  AAA-TTTCTTT-ATCTTTG---TCTAC--TA-T---TCGG-GGAATTAATTAACACATGGCTGCACAAAGCAAAGTTTTTCTTTTTGTCATAATGAACAAA 286

     283  TCTTTGATTCATGCTTTATGCGGTTGATCATGAAAAAA-CATTCATGTTGTTCATATTAATTATGTCGCACTTAAACGAAGATCTATAATTAAGACTTTT 381
     287  TCTTTGACTCATGCTTTATGCGGTTGTTCATGAAAAAAACATTCATGTTGTTCATATTAATTATGTCGCACTTAAACGAAGATCTATAATTAAGGCTTTT 386

     382  ACTTTCA-CTGACAAAGCGAAAAGATACCAATAATTTTTTTG-GAC-TGTCCTTTAGTACATGAATTCAATGACATTTTCTGTACGAGCACGTGTCTTTG 478
     387  ACTTTC-GCTGACAAAGCGAAAAGATACCAACAATTTTTTT-CGA-TTGTCCTTTAGTACATGAATTCAGTGACATTTTCTGTACGAACACGTGTCTTTG 483

     479  TGTGGCAATAATT-ATATATAATTTCTGTTAGTGTGATTCTTCGTGATAAAATTATTGGTTTTGTAGGGCTTAATAGGTGTAGGAAGAGTTGTAGACTAA 577
     484  TGTGG-A--AAT-AATATATAATTTCTGTTAGTGTTATTCTTCGTGATAAAATTATTGGTTTTGTAGGGCTTAATAGGTGTAGGAAGAGTTGTAGACTAA 579

     578  GATGGCTGAACTATTTGAAGCCAACTATCAAGAGAGGAAAACTTAGCTCTGATGAAGTTGATCTTCTTCTCCGTCTTCATAAGCTTTTAGGAAACAGGTT 677
     580  GATGGCTGAACTATTTGAAGCCAAATATCAAGAGAGGAAAACTTAGCTCTGATGAAGTTGATCTTCTTCTCCGTCTTCATAAGCTTTTAGGAAACAGGTT 679

     678  TGTATTCTTAAGACAAAAATTCAACTTTGTT-TCTTGTCTAATGATCCATAAGA--------------TATATATATGTATATCCAAATCGTTCAAATGC 762
     680  TGTATTCTTAAGACAAAAATTCAACTT-GT-ATCTTGTCTAATGATCCATAAGATATATATATATATATATATATATGTATATCCAAATCGTTCAAATGC 777

     763  ATGCTTAGGTGGTCTTTAATTGCTGGTAGACTACCCGGTCGGACCGCTAATGATATCAAGAATTACTGGAACACCCATCTGAGCAAGAAACATGAACCAT 862
     778  ATGCTTAGGTGGTCTTTAATTGCTGGTAGACTACCCGGTCGGACCGCTAATGATATCAAGAATTACTGGAACACCCATCTGAGCAAGAAACATGAACCAT 877

     863  GTTGTAAGACCAAGATGAAGAAGAGAAACGTTACATTCTCTTCTACCACACCCGCCCAAAAAATCGACGTTTTCAAACCTCGACCTCGACTCTTCACCGT 962
     878  GTTGTAAGACCAAGATGAAGAAGAGAAACGTTACATTCTCTTCTACCACACCCGCCCAAAAAATCGACGTTTTCAAACCTCGACCTCGACTCTTCACCGT 977

     963  TAACGATGGCTGCAGCCATCTCCATGGCCTGCCAGAAGTTGACGTTGTTCCTCCATGCGTTGGACTCAACAACATTAATAATGTCTGTGAAAATAGTATG 1062
     978  TAACAATGGCTGCAGCCATCTCCATGGCCTGCCAGAAGTTGACGTTGTTCCTCCATGCCTTGGACTCAACAACATTAATAATGTCTGTGAAAATAGTATG 1077

    1063  ACATGTAACAAAGCTGGGGAGAAGTATGAACTTTTTAGTAATTTAATGGATGGAGAGAATATGTGGTGGGAGAGTTTGCTAGAGCAGAGCAAACAGCCTG 1162
    1078  ACATGTAACAAAGCTGGGGAGAAGTATGAACTTTATAGTAATTTAATGGATGGAGAGAATATGTGGTGGGAGAGTTTGCTAGAGGAGAGCAAACAGCCTG 1177

    1163  ACGGGCTCGTTCCAAAAGGTACGGCAACAAAAAAGGGGGCAACCTTTGCGTTTGACGTTGAGCAACTTTGGAATATGTTGGATGGAGAGACTGTAGAACT 1262
    1178  ACGGGCTCGTTCCAAAAGGTACGGCAACAAAAAAGGGGGCAACCTTTGCGTTTGACGTTGAGCAACTTTGGAATATGTTGGATGGAGAGACTGTAGAACT 1277

    1263  TGATTAG 1269
    1278  TGATTAG 1284
```

Supplemental Figure S1C. Nucleotide sequence alignment of R500 (upper) and L58 (lower) alleles of the *PAP2(A03)* (BraA03g40160R) gene. Exonic sequences are highlighted in cyan and intronic sequences are not highlighted. Nucleotide polymorphisms are indicated by red, with substitutions indicated by # and indels indicated by filled boxes. Asterisks mark 10 bp intervals.
