# Supplementary material for: Genetic and genomic resources to study natural variation in Brassica rapa
Source: Plant Direct. 2020 Dec 22;4(12):e00285. doi: 10.1002/pld3.285 (PMC7755128; doi:10.1002/pld3.285)
Supplement: Supplementary file 2 — Fig S2 [file PLD3-4-e00285-s002.pdf]

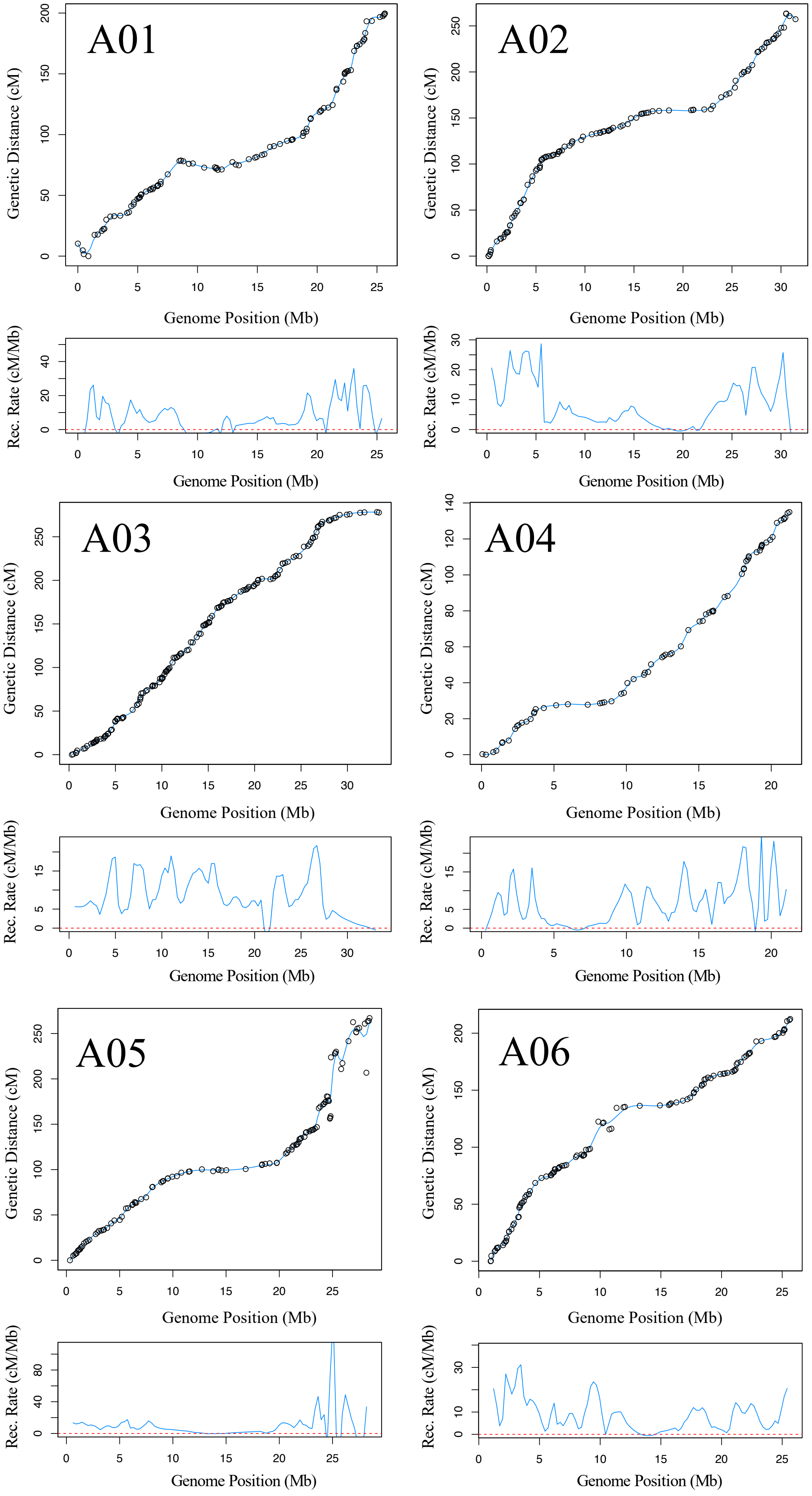
A01
Genetic Distance (cM)
Genome Position (Mb)
Rec. Rate (cM/Mb)
Genome Position (Mb)
A02
Genetic Distance (cM)
Genome Position (Mb)
Rec. Rate (cM/Mb)
Genome Position (Mb)
A03
Genetic Distance (cM)
Genome Position (Mb)
Rec. Rate (cM/Mb)
Genome Position (Mb)
A04
Genetic Distance (cM)
Genome Position (Mb)
Rec. Rate (cM/Mb)
Genome Position (Mb)
A05
Genetic Distance (cM)
Genome Position (Mb)
Rec. Rate (cM/Mb)
Genome Position (Mb)
A06
Genetic Distance (cM)
Genome Position (Mb)
Rec. Rate (cM/Mb)
Genome Position (Mb)

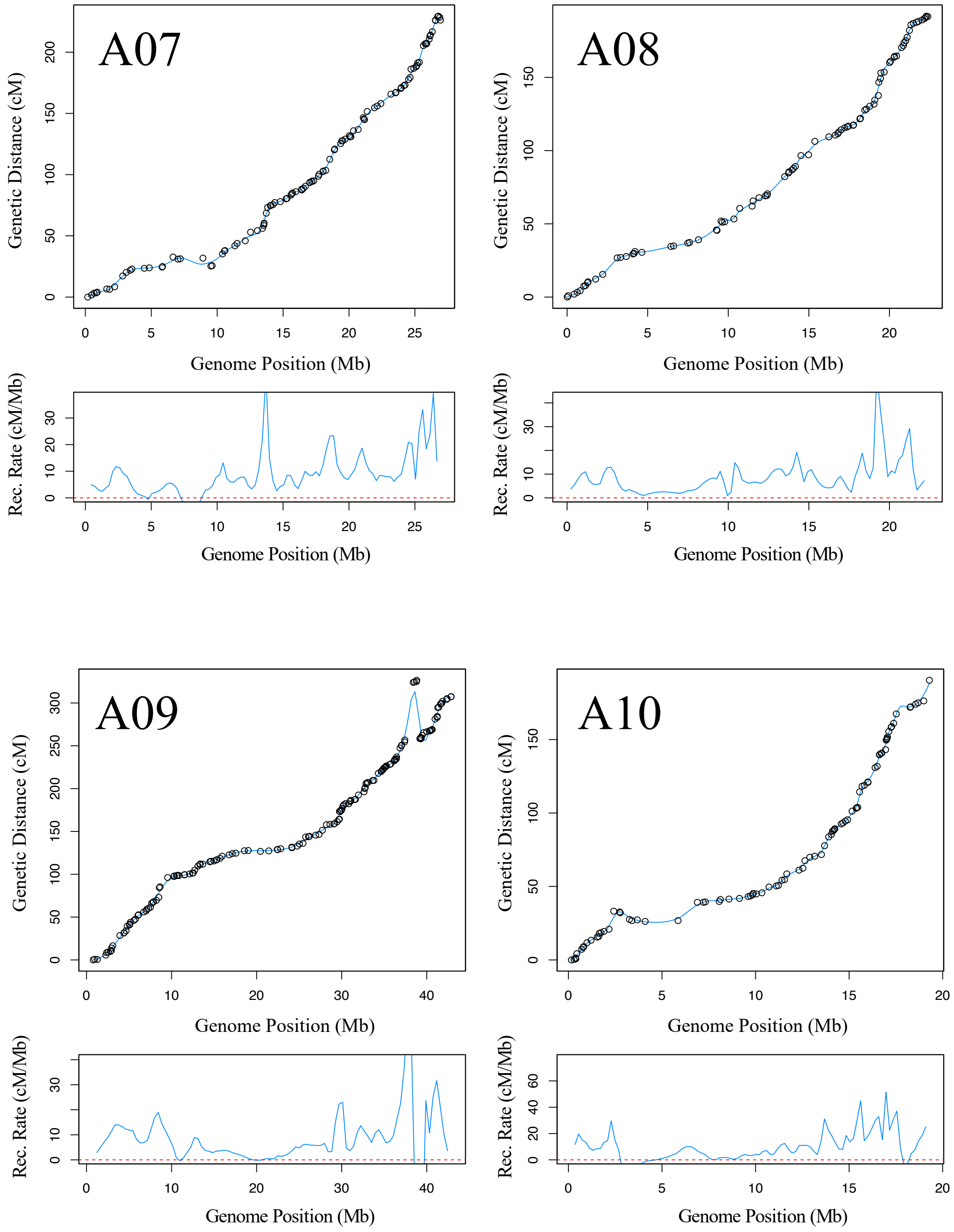

Supplemental Figure S2. The correlation of genetic and physical distance, estimated local recombination rates (Rec. Rate) and its distribution in *Brassica rapa* R500 x L58 Population in chromosomal rearrangement regions. The black dots represent the genetic and physical positions of SNPs, blue curve below represent the estimated local recombination rates.
