# Supplementary material for: Genetic and genomic resources to study natural variation in Brassica rapa
Source: Plant Direct. 2020 Dec 22;4(12):e00285. doi: 10.1002/pld3.285 (PMC7755128; doi:10.1002/pld3.285)
Supplement: Supplementary file 3 — Fig S3 [file PLD3-4-e00285-s003.pdf]

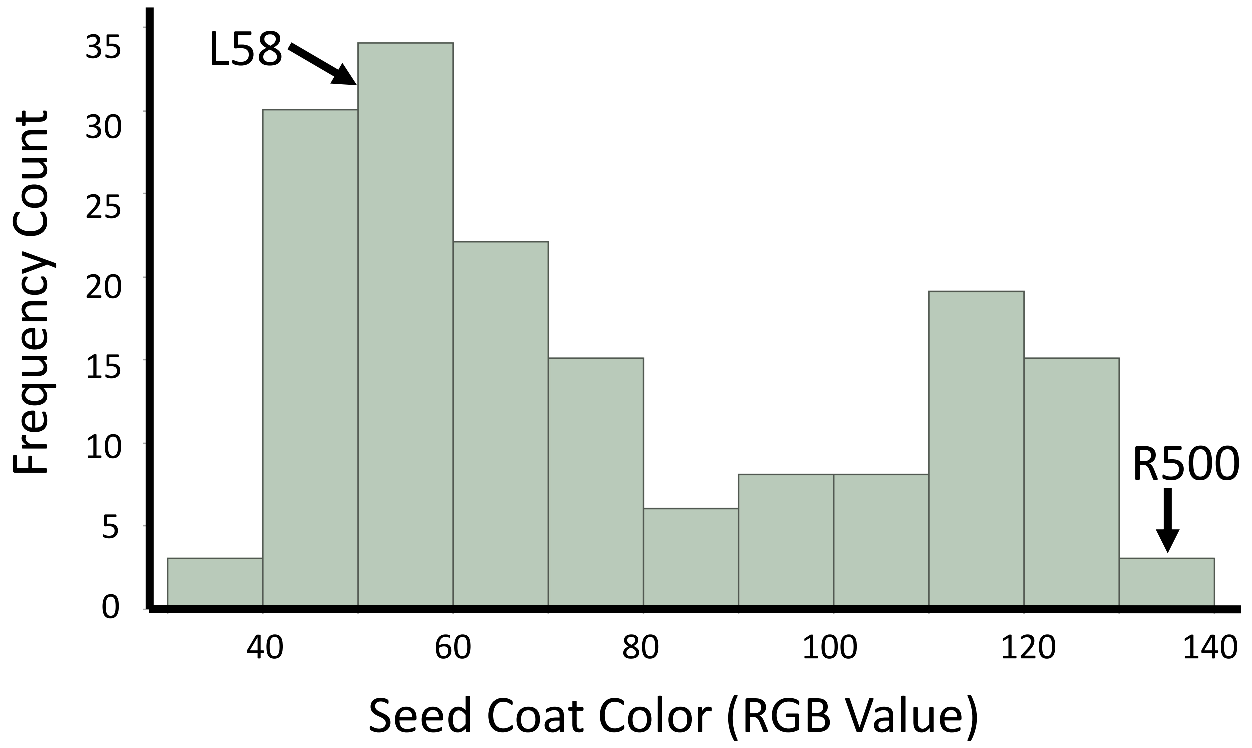

Supplemental Figure S3. Distribution of seed coat color among the AI-RIL population derived from a cross of R500 x L58. Parental values are indicated. Lower RGB values indicates darker seeds and higher RGB value indicates yellower seeds.
